# Attitudes of Jordanian Adolescent Students Toward Overweight and Obesity

**DOI:** 10.2174/1874434601812010015

**Published:** 2018-01-31

**Authors:** Nesrin N. Abu Baker, Nahla Al-Ali, Ranyah Al-Ajlouni

**Affiliations:** 1 Faculty of Nursing, Jordan University of Science and Technology, Irbid 22110, Jordan; 2 Princess Salma Faculty of Nursing, Al-Albayt University, Mafraq 2, Jordan

**Keywords:** Adolescents, Attitudes, Obesity, Overweight, Health program, Jordan

## Abstract

**Background:**

Obesity is a serious public health problem especially among adolescents. Understanding adolescents’ attitudes toward obesity and healthy lifestyle is a crucial step to develop effective health programs to treat and prevent obesity.

**Objectives:**

To examine the attitudes toward overweight and obesity among Jordanian adolescent students and to identify the components of obesity prevention program that the students perceive as important.

**Methods:**

A sample of 1000 students in 8^th^ to 10^th^ grades was randomly selected from 16 schools in Irbid, Jordan. A self-reported questionnaire including attitude related questions was used in a descriptive, cross-sectional study.

**Results:**

Generally, the students expressed positive attitudes toward obesity; which means that their attitudes were consistent with societal norms in terms of health and social functioning (mean= 3.5, SD=0.39). Furthermore, the students expressed positive attitudes toward lifestyle; which means that their attitudes were consistent with healthy behaviors (mean=3.7, SD=0.58). However, boys had significantly more positive attitudes than girls *(p*=0.04). The prevalence of overweight and obesity was 23.8%, while obese and non-obese students had similar attitudes toward lifestyle and obesity. Finally, around 20% to 30% of students desired a prevention program out of school time shared with their families and friends and involves eating healthy food and getting more exercise.

**Conclusion:**

More efforts are needed to build effective obesity prevention programs that focus on eating healthy diet and getting more exercise considering gender differences.

## 1 INTRODUCTION

Obesity has more than doubled since 1980 Worldwide, at least 2.8 million people die each year as a result of being overweight or obese [1]. The trends are particularly concerning in the Middle East where, in addition to the reported trends in the adult population obesity is a serious health concern for children and adolescents as well [2, 3]. Middle Eastern researchers reported upward trends in child and adolescent obesity over the past decade with the CASPIAN III study reporting “alarming evidence-based data on the considerable prevalence of obesity, metabolic syndrome and cardiovascular disease risk factors in the adolescent age group” [4]. This growing problem requires government action on the primary level of prevention [2].

According to the World Health Organization (WHO), the major causes that lead to obesity during childhood or adolescence are increased intake of energy-dense food, decreased physical activity, sedentary lifestyle, changing modes of transportation, increasing urbanization and other societal reasons [5]^.^ However, attitudes toward obesity affect adolescents’ lifestyle. Attitudes in general are formed over a lifetime through an individual’s socialization process that includes one’s own values and beliefs during childhood years. Children’s and adolescents’ attitudes factors such as parental attitude, school, governmental policy and socioeconomic status [6].

The objectives of this study were to (1) examine attitudes toward overweight and obesity among Jordanian adolescent students in 8^th^ to 10^th^ grade; (2) to compare students’ attitudes toward overweight and obesity between adolescent students who are obese and non-obese. (3) examine differences of attitudes toward overweight and obesity among students with different levels of socio-demographic data (gender, age, living area, household income, parents’ educational level, parents’ job, and type of school); and (4) examine students’ perception of the components of obesity prevention program).

### 1.1 Significance of Study

Many researchers focused on investigating the prevalence of obesity and its associated factors and consequences. However, little researchers investigated adolescents’ attitudes toward these problems especially in the Arab countries. Even these studies are lacking in Jordan. It is essential to examine the attitudes toward obesity and healthy lifestyle among adolescents since obese adolescents with family history of obesity have a great potential to suffer from obesity and its consequences in future. Thus, understanding adolescents’ attitudes toward obesity and healthy lifestyle is a crucial step to develop effective health programs to treat and prevent obesity among adolescents. These adolescents are the parents, workers, and leaders of future and their success depends on their health status.

## 2 LITERATURE REVIEW

Only few studies investigated adolescents’ attitudes or their parents’ attitudes toward obesity especially in the Arab countries. In Jordan, the prevalence of overweight and obesity had increased among children and adolescents in the last three decades. A study showed that the highest prevalence of overweight and obesity was reported at age 15 (24.4% and 8.9%, respectively) [7]. It is assumed that Jordanians’ attitudes might be affected with the dramatic changes in lifestyle. Such lifestyle is characterized by the dependency on transportation; increasing the consumption of fast food and sweets. In addition, leisure time became dominated by viewing TV, playing computer games, and using internet [8].

In most previous studies, children and adolescents perceived obesity as a health issue that can be promoted through healthy lifestyle. For example, a mixed methods approach was used to assess attitudes towards obesity among 159 parents and 184 of their children (aged 9–18 years old) in Australia. A face to face interview was used. The results revealed that two clusters (27% and 38%) of the sample identified obesity as a serious health problem. Participants thought that lifestyle and environment were the major cause of obesity. They also preferred positive messages (including healthy eating and activity) targeting individual to encourage people to lose weight [9]. On the other hand, a cross-sectional study was conducted in randomly selected primary schools in Tanzania. A structured questionnaire was used to assess the knowledge and attitudes of 446 children toward obesity. Most children had correct attitudes towards obesity during childhood. More than two-thirds (67.5%) of the children disagreed that it is good for a child to be or look obese. Majority (85.0%) of the children agreed that children should do physical activities and (77.1%) disagreed that obesity is an indicator of good health. Regarding prevention, only one-third were aware of the ways to prevent childhood obesity. Regarding knowledge about risk factors for obesity, less than half of the children (45.4%) had knowledge about the risk factors [10].

Another study was conducted in Poland to find an association between health awareness in children and the risk of becoming overweight or obese. A sample of 1,515 healthy children aged 6-18 years was included. A questionnaire about diet habits and physical activity was also used. The results revealed that girls chose food products that they believe to keep them fit significantly more than boys, while boys spent more time in front of a computer or TV than girls. The authors of this study concluded that awareness of healthy lifestyle behavior is not enough to maintain optimal body mass. Knowledge about proper eating habits is better among girls than among boys, especially in the older age groups [11].

The investigation of the adolescents’ attitudes and behaviors toward obesity became a necessary step in developing effective health promotion programs to prevent overweight and obesity among adolescents [12]. This has influenced lead nurses and other health professionals throughout the world to give more concern and attention to the attitudes and behaviors impacting the problem of overweight or obesity [13]. Therefore, health promotion strategies based on multi-component interventions (including local government, advertising and media, schools and home) are recommended to enhance adolescent’s knowledge and attitudes [14].

### 2.1 Research Design and Setting Methods

A descriptive, cross sectional survey design was used for the purpose of this study. This study was conducted in the northern part of Jordan, in the educational directorates of Irbid governorate. Irbid is the second largest city in Jordan with a total population of 1,775,200 [15]. Irbid area is divided into seven directorates.

### 2.2 Sample and Sampling Method

A multistage, random sample was used to recruit the study sample. At first stage, a list of all governmental schools was obtained from educational directorates in Irbid governorate. In the second stage, two lists were created by the primary investigator for male and female schools, and then two schools were selected randomly from each directorate, one for females and another for males. In the third stage, three classes (8^th^, 9^th^, and 10^th^) were selected randomly from each selected school. Finally, all eligible students in each selected class were voluntary involved in the study. Also, a list of private schools was created, and then one school was selected randomly from each directorate.

The inclusion criteria consisted of all Jordanian students affiliated in educational directorate in Irbid city, who are mentally and physically healthy, and who have consent from their families. Students who have growth problem, on hormonal therapy or on medication with side effects of increasing body weight were excluded from participating in this study. Data about the inclusion and exclusion criteria were assessed from student’s medical charts in their schools.

The total sample size was calculated based on the following formula: N= [(1.96)^2^ * pq]/d^2^. Where: N= sample size, P= expected prevalence, Q= 1-p, D= margin of error. To calculate 95% confidence interval for p that is expected to be 25% (based on the average percentage of prevalence of overweight and obesity in Jordan) with the margin of error (d) not more than 0.03, the needed sample size was 800. N= (1.96)^2^ * (0.25) * (1-0.25) / (0.03)^2^= 800. To cover the drop out of any participant or missing data, the researcher enlarged the sample. Thus, 1060 questionnaires were distributed, but 1000 questionnaires were returned back with a response rate of 94%.

### 2.3 Measurement

Self-reported questionnaire and anthropometric measurement were used to collect data in this study. A modified version of the Attitude of Adolescents’ toward Obesity Questionnaire was adopted from Wilson [16] to collect the data about students’ attitudes toward overweight and obesity. Firstly, the original items that measure attitudes were modified from multiple choice questions (e.g. “What is your opinion towards keeping a healthy weight? Check all that apply; In order to have a healthy balance between calories eaten and calories used up, which do you think would work best for you? Check all that apply” [16]) to Likert-type scales. Secondly, open ended questions (“When you see someone who is overweight what do you think about that person, what goes through your mind? What kinds of problems do overweight or obese students have at School?” [16]) were modified to be close-ended questions. Thirdly, one question remained the same (“If you were to participate in some type of activity/program to help you be healthier, which would you prefer?” [16]). Fourthly, few items were added or deleted based on the experts’ review. Finally, items about socio-demographic data and body mass index were also added.

The instrument used in the current study was a self-reported questionnaire that was consisted of two sections. The first section of the instrument included socio-demographic data which consisted of 20 items (such as age, grade, gender, living area, educational directorate, parents’ job and education level). The last three items asked about weight, height, and BMI, which was filled by the researcher based on anthropometric measurement; body weight was measured by the researcher with a digital scale to the nearest 0.1 kg in light clothing and with bare feet or stockings, and height was measured by a wall-mounted stadiometer without shoes and recorded to the nearest 0.5 cm. These measures were used to calculate the Body Mass index (BMI) according to the following formula: BMI = weight in kilogram/ (height in meter) ^2^.

The cut-off points for detecting weight status were taken from Centers of Disease Control and Prevention (CDC) growth chart from 2 -20 years old. The CDC use the percentile of body mass index to assess overweight and obesity among children and adolescents. That is 85^th^ percentile of body mass index as a cut off point for overweight, and the 95th percentile as a cutoff point of obesity among children and adolescents [17].

The second section consisted of three questions that measured the students' attitude toward lifestyle affecting weight status, and toward obesity as a problem, and to identify the components of a health program targeting students to prevent or treat obesity.

The scale of the first question contained ten items about the attitude of students toward certain lifestyle behaviors to prevent obesity. The responses to the Likert-type scale questions were answered on a 5-point Likert-type scale ranging from 1 (not very important) to 5 (very important) where the higher the score, the more positive the attitude is to maintain healthy weight. However, two items (7 and 8) were reversed.

The second question contained 12 items. These items were all Likert-type scale, in which 5 responses ranged from 1 (strongly disagree) to 5 (strongly agree), the items assess students’ attitudes toward obesity, prevention strategies, and health-related consequences. Responses to all questions were answered on a 5-point Likert-type scale ranging from 1 to 5, where 1 indicates strongly disagreement and 5 indicates strongly agreement. The higher the score, the more positive the attitude is toward obesity prevention and health-related consequences. However, three items (2, 4, and 9) were reversed

The third question was to detect the components of the obesity prevention program that the students perceive as important. It was a multiple choice question with seven options, from which the student can check more than one.

### 2.4 Validity and Reliability of the Instrument

Because the native language in Jordan is Arabic, the new modified instrument was translated from English to Arabic. Then, the instrument was back translated from Arabic to English by a person who knows both languages very well. In addition, a panel of three experts in the Faculty of Nursing of Jordanian universities examined the content validity of the instrument.

After that, a pilot study was conducted to examine the reliability of instrument, to estimate the time needed to complete the questionnaire, and to identify the practical limitations of the instrument. The pilot was conducted in two governmental schools in Irbid (one for girls and the other for boys). Three classes (8^th^, 9^th^, and 10^th^ grades) were randomly selected from each school, eighty participants completed the questionnaire. The time to fill the questionnaire was appropriately 15 minute for each student. The students reported that the questions were simple and easy to understand. The Cronbach's alpha was 0.80 for the whole instrument. Cronbach's alpha was 0.72 and 0.64 for lifestyle and obesity, respectively.

### 2.5 Data Collection and Ethical Consideration

Ethical approval was obtained from the Scientific Research Committee at the Faculty of Nursing and the Institutional Review Board (IRB) at Jordan University of Science and Technology. Permission was obtained from all Irbid directorates after the approval from Ministry of Education.

The study was conducted in September, 2011. The researcher met the head of school, and explained the purpose of the study and permission was taken to see students in their classrooms, and to access the medical files of students who agree with their parents to participate. The purpose of study and procedures were also explained to all students and the students were assured that they have the right to participate or refuse to participate. Then, an informed consent was signed by adolescent’s parents at home before data collection. Students whose parents signed the consent form were included in the study and implied consent was assumed if participants voluntarily completed the questionnaire. After that, the questionnaire was administered and explained to the students. Then, the student’s weight and height were taken and recorded by the researcher after the student’s completion of the questionnaire.

All the students who completed the questionnaire were eligible to the study based on medical records. A code number was assigned and used instead of a name on all data collection tools. After the data were collected from the participants, the link between the code number and the participant's name was destroyed. To maintain confidentiality, students were assured that all the information that was obtained will be kept secret. Furthermore, students were assured that no other person will know their personality, and data was coded to assure anonymity. The data were kept in locked files separately from any participant’s name or identifiable information.

### 2.6 Data Management and Analysis Plan

Descriptive statistics such as (means, standard deviations, frequencies, and percentages) were used to synthesize and describe data. Additional bivariate statistics such as independent t-test and Pearson correlation coefficient were used to answer the research questions.

## 3 RESULTS

### 3.1 Socio-Demographic Characteristics of the Sample

The total sample in this study was 1000 students; sixteen schools were included representing 8^th^, 9^th^, and10^th^ grades in all schools Irbid Governorate. Each grade composed around one third of the sample; 35.6% (n= 356) were 8^th^ graders, 34.1% (n= 341) were 9^th^ graders, and 30.3% (n= 303) were 10^th^ graders. The age of students ranged between 13-15 years (mean=13.98, SD= 0.81). Other demographic characteristics are shown in Table **1**.

The overall prevalence of overweight and obesity was 14.9% and 8.9%, respectively (23.8%). About 7.3% and 5.5% of males had overweight and obesity respectively, and 7.6% and 3.4% of females had had overweight and obesity respectively.

### 3.2 Students’ Attitudes toward Lifestyle and Obesity

*Student’s attitude toward lifestyle*: The students’ attitudes toward lifestyle affecting weight status were considered positive (consistent with healthy behaviors) if the mean score of each item was more than 3 and negative if the mean score was less than 3. The study results revealed that the students expressed positive attitudes toward lifestyle (mean=3.7, SD=0.58). For example, they expressed positive attitudes toward getting more exercise, eating less junk food or sweets and drinking more water. On the other hand, the students had negative attitudes (inconsistent with healthy behaviors) toward using medication that help in reducing weight and toward depending more on transportations as shown in Table **2**.

*Students’ attitude toward obesity as a problem*: The study result revealed that the student expressed positive attitude (consistent with societal norms in terms of health and social functioning) toward obesity (mean= 3.5, SD=0.39). For example, they expressed positive attitude toward the statement “it is unhealthy to be overweight weight”. Also they had positive attitude toward the statement “overweight people should try to lose weight”. On the other hand, the students expressed negative attitude (inconsistent with societal norms in terms of health and social functioning) toward statements such as “it is okay to be overweight” and “obesity among young people is a manifestation of manhood and prestige that enhance other’s respect” as shown in Table **3**.

### 3.3 Differences in Attitudes between Obese and Non-obese Students

Independent sample t-test was conducted to investigate the difference in attitudes between obese and non-obese students. The study results showed that there was no statistically significant difference between adolescent students who are obese (mean=4.07, SD=0.66) and those who are non-obese (mean=4.1, SD=0.65), in term of attitude toward lifestyle *t* (998) = 0.48, *p*= 0.63. Also the results showed that there was no statistically significant difference between adolescent students who are obese (mean=4.0, SD=0.45) and those who are non-obese (mean=4.0, SD=0.41), in term of attitude toward obesity *t*(998)=0.05, *p*=0.96.

### 3.4 Differences of Attitudes among Students with Different Levels of Socio-demographic Data

Independent sample t-test was conducted to investigate the differences of attitudes and behaviors toward obesity among students with different levels of socio-demographic data (gender, sector of school, parents’ job, and parents’ educational level). The study results showed that there was a statistically significant difference between male students (mean=4.05, SD=0.66) and female student (mean=4.14, SD=0.63), in term of attitude toward lifestyle *t* (998)=2.1, *p*=0.04. However, there was no statistically significant difference among students with different levels of socio-demographic factors toward life style or toward obesity *p* > .05 as shown in Table **4**.

A Pearson correlation coefficient was computed to assess the relationship between the attitudes and students’ age and students’ family monthly income. There was no significant correlation between student’s age and students’ attitude toward lifestyle [r= -0.36, n=1000, *p*=0.26]. In addition, there was no significant correlation between student’s family monthly income and students’ attitude toward lifestyle [r=-0.017, n=1000, *p*=0.60]. The result also indicated that there was no significant correlation between age and students’ attitude toward obesity [r=0.032, n=1000, p=0.31]. However, there was only a significant positive correlation between family monthly income and students’ attitude toward obesity as a problem [r= 0.064, n=1000, *p*= 0.04].

### 3.5 The Components of the Obesity Prevention Program that the Students Perceive as Important

Based on multiple responses per each student, around 31% of students desired a prevention program out of school time shared with their families and friends. Moreover, about 25% of the students preferred a program that involves eating healthy food. While 20% preferred attending the program by themselves out of school and getting more exercise. These components and other components of this prevention program are shown in Table **5**.

## 4 DISCUSSION

### 4.1 Students’ Attitudes toward Lifestyle and Obesity

The first purpose of this study was to examine students’ attitudes toward obesity, the results showed that the students had positive attitude toward healthy lifestyles such as getting more exercise especially in schools, and eating healthy food including fruits and vegetables, drinking more water, and watching programs about obesity prevention. On the other hand, the students had negative attitude toward unhealthy lifestyles such as playing video/computer or watching TV, depending on transportation, eating sweet or junk food, and drinking soda. These results are consistent with the previous study conducted by Olds *et al.* [9] in Australia to assess attitudes towards obesity among parents and their children, where they thought that lifestyle is a primary cause of obesity which is considered a serious health problem. Likewise, the study that was conducted by Njelekela [10] in Tanzania found that the majority of school-children agreed that they should do physical activities and disagreed that obesity is an indicator of good health. However, only one-third were aware of the ways to prevent childhood obesity. An explanation for these positive attitudes toward obesity might be the cumulative role of family, school, and widespread media in shaping these attitudes early.

In addition, the students considered it is hard to keep a healthy weight, but it becomes easier with family support and if the students earning a prize, also they agreed that school plays an important role to help students who need to treat or prevent obesity. These results are similar to those in the previous study which concluded that adolescents reported that school, families, and friends were major influences on students’ attitudes and behaviors to keep healthy weight; it may be because the families and teachers are the role models for them [18]. Corresponding with this result, the study which found that parents and their children also preferred key messages such as healthy eating and physical activity to target individual’s choice as the best way to get people to lose weight [9].

### 4.2 Differences of Attitudes among Students with Different Levels of Socio-demographic Data and Obesity Status

The current study explored the differences in students’ attitude by gender, parent’s job, parents’ level of education, school sectors, and status of obesity. The study results showed that male students had significantly more positive attitudes than female students toward life style. However, the study that was conducted by Olds *et al.* [9] found that the cluster (35% of the sample) which did not consider obesity as a serious issue tends more likely to be males. In addition, another study found that girls significantly more frequently chose food products that are believed to keep them fit, while boys spent more time in front of a computer or TV than girls [11]. While explaining the study results, it should be taken in consideration that culture plays a vital role in shaping the gender difference between males and females with regard to attitudes especially toward lifestyle activities.

The results of this suggested that all students who were obese and who were non-obese had similar attitudes toward lifestyle affecting weight status and toward obesity. Although some studies indicated that BMI was a significant factor for perception of weight [12]. In this study, having a sample that represents only a certain age group in a in a certain part of the country might explain the difference in the results. In addition, since the prevalence of overweight and obesity had increased in Jordan among children and adolescents in the last three decades, it is assumed that their attitudes have been affected accordingly.

The current study showed that no differences were found in students’ attitudes toward lifestyle and obesity with different levels of socio-demographic data; parents’ job, and parents’ level of education. It might be explained by the weak relationship between students and their parents and decreasing the influence of role modeling of parents. In addition, adolescents are affected by media advertisements more than parents, beside that most adolescents now prefer fast food more than homemade food even if their mothers are unemployed.

According to the school sectors, no differences were found between students’ attitudes, this may be related to the fact that all types of schools sell fast and sweet food inside or surrounded by supermarkets or restaurants outside. Another factor might be due to the small sample size of private schools compared to governmental schools in Irbid governorate. Furthermore, there were no differences in attitudes between students who live in rural or urban areas. It may be explained by the fact that nowadays, all public services such as transportation, different types of food and media are available in rural and urban areas in Jordan.

### 4.3 The Components of the Obesity Prevention Program that the Students Perceive as Important

Previous studies have promoted a school-based intervention program shared with friends, it may be due to adolescents’ natural need for socialization [16], but the students in this study preferred a program out of school that is shared with their family. A study concluded that physical activity with family members can improve health status for adolescents because the parents are their role models, but the family usually do the exercise out of family time so the adolescent cannot share them [19]. Forty two intervention studies were reviewed; the interventions consisted of nutrition, physical activity, and behavior education or combination of these activities. The results revealed that parent participation significantly predicted intervention effectiveness, *p*= 0.001 [20].

In addition, according to students’ responses in the current study, the effective obesity prevention program involves eating healthy food more than getting more exercise, it may be due to that the student are unable to make control alone on their eating habits by decreasing the amount of unhealthy food, because it is available in all places, but they can alone increase their physical activity to make calorie balance, so they prefer to focus more on healthy diet in obesity prevention programs.

### 4.4 Practice Implications

The findings of the present study should be used as an assessment study to develop a comprehensive obesity prevention program at all levels; individual, family, school, and community, especially in schools where children and youths spend a significant amount of time. School nurses should educate school staff including administers of school, teachers, and school nurses about healthy eating habits and the benefit of physical activity. School staff needs to collaborate with policymakers, health care providers and Ministry of Health in order to develop effective intervention. This intervention should improve physical activity by increasing the number of sport classes and improving healthy eating habits among students.

Health professionals can benefit from the current study findings by educating students about the prevention and treatment of obesity across the lifespan to prevent or reduce the chronic diseases in adulthood. Integrating educational programs early in primary schools is an effective strategy to impact knowledge about obesity diseases early in childhood [10]. Including family members, especially parents is also essential, taking in consideration the context in which adolescent fits into family structure [21].

### 4.5 Recommendations

Based on the findings of the current study and the result published elsewhere in literature; future research is needed in longitudinal studies at the national level not only in the northern part of Jordan. The purpose of future research is to build effective intervention strategies for obesity prevention program conducted in various settings, particularly in the schools and primary care settings that can compare and evaluate the interventions. Also, the obesity prevention program should involve students’ parents and friends.

Authors of the previous studies recommended several approaches to improve these prevention programs. For example, family functioning may be protective for adolescent weight and weight-related health behaviors [22]. Furthermore, programs addressing eating behavior and physical activity for children and adolescents can maintain a healthy weight [23]. Parental interventions including communication and motivation of adolescents regarding weight-related topics, appropriate autonomy, and addressing negative emotions can be effective [21]. Finally, conducting a qualitative study is recommended to ask obese children about their life style behaviors in order to achieve in-depth assessment for their attitudes.

#### 4.5.1 Limitations of the Study

In the current study, the sample consisted of adolescents studying in 8^th^ – 10^th^ grades, which make it hard to generalize the results to adolescents of other grades. The sample was not representative to Jordanian students as a whole because this study represents only the northern part of Jordan. On the other hand, the private schools are available only in two directorates which make it difficult to compare the students’ attitude by schools in all directorates.

## CONCLUSION

The study revealed positive attitudes among students toward lifestyle affecting weight status and toward obesity as a health problem. However, the prevalence of obesity and overweight is still high, this indicates that more efforts are needed to build effective obesity prevention programs that focus on eating healthy diet and getting more exercise. In addition, the results did not reveal significant differences among students with different socio-demographic data or obesity status, which indicate that the programs should be directed toward all levels of schools and students.

## Figures and Tables

**Table 1:** Socio-demographic characteristics for the study sample.

**Variable**	**Frequency (n)**	**Percentage (%)**
**Gender**		
Male	520	52.00%
Female	480	48.00%
**Living Area**		
Rural	503	50.30%
Urban	497	49.70%
**Parent’s Educational Level**		
Equal or less than high school		
Father	569	56.90%
Mother	555	55.50%
More than high school		
Father	431	43.10%
Mother	445	44.50%
**Father’s Job**		
Private or Vocational Sectors	513	51.30%
Governmental or Military Sectors	487	48.70%
**Mother’s Job**		
Employed	182	18.20%
Unemployed	818	81.80%
**School sectors**		
Private	183	18.30%
Governmental	817	81.70%

**Table 2:** The mean and standard deviation of students’ attitudes toward lifestyle.

**Item**	**(Mean ±SD)**
1. Get more physical exercise.	4.4 ± (0.88)
2. Play less video/computer games and watch less TV.	3.5 ± (1.17)
3. Eat less junk food or sweets	4.4 ± (0.88)
4. Eat more fruits and vegetables.	3.8 ± (1.17)
5. Drink more water.	4.4 ± (0.91)
6. Drink less soda	3.6 ± (1.28)
7. Depend more on transportations, especially to school	2.9 ± (1.48)
8. Using the medication that help in reducing weight	2.3 ± (1.37)
9. Watch programs about obesity; prevention and consequences	3.5 ± (1.39)
10. Participate in exercise programs in schools or sport clubs	3.9 ± (1.20)
**Total students’ attitude toward lifestyle**	**3.7 ± (0.58)**

**Table 3:** The mean and standard deviation of students’ attitudes toward obesity.

**Item**	**(Mean ±SD)**
1. It is unhealthy to be overweight.	4.4 ± (1.03)
2. It is okay to be overweight.	1.9 ± (1.11)
3. Overweight people should try to lose weight	4.5 ± (0.91)
4. It is hard to keep at a healthy weight	3.6 ± (1.18)
5. It is easier to eat healthy and exercise with family support/ help	4.3 ± (0.95)
6. Earning a prize facilitate eating fruits and vegetables, get more exercise, and spend less time playing video/ computer games.	3.9 ± (1.04)
7. Obesity makes people isolated from others because of their appearance	2.7 ± (1.29)
8. Obesity reduces self-esteem and self-confidence	2.9 ± (1.30)
9. Obesity among young people is a manifestation of manhood and prestige that enhance other’s respect	2.6 ± (1.35)
10. Maintaining ideal body weight is a goal to improve the external appearance only	3.4 ± (1.36)
11. School should help students who are already overweight or who want to prevent themselves from becoming overweight	4.2 ± (0.99)
12. I am personally interested in eating healthy and getting enough exercise	4.1 ± (1.05)
**Total students’ attitude toward obesity**	**3.5 ± (0.39)**

**Table 4:** The differences of attitudes toward lifestyle and obesity among students with different levels of socio-demographic data.

	**Attitude toward lifestyle**			**Attitude toward obesity**		
**Variable**	**t**	**df**	**P**	**t**	**df**	**P**
**Gender**	2.1	998	0.04	0.67	987.6	0.5
**School sector**	1.03	998	0.29	2.22	998	0.26
**Parents’ job**						
father	0.4	998	0.69	0.48	998	0.63
mother	0.82	998	0.93	1.23	998	0.22
**Parents’ educational level**						
father	0.18	998	0.86	1.32	998	0.19
mother	0.21	998	0.83	1.61	998	0.1
**Living area**	0.12	998	0.91	1.13	998	0.26

Note: P-value less than 0.05

**Table 5:** The components of preferences of obesity prevention program.

**Rank**	**Item**	N (%)*
1	A program away from the school not during school time.	314 (31.4)
2	A program my family would also participate in	314 (31.4)
3	A program involving a group of student/ teenagers.	289 (28.9)
4	A program involving learning how to eat healthy food.	245 (24.5)
5	A program showing me how to get more exercise.	200 (20.0)
6	A program that I would attend by myself as in individual.	192 (19.2)
7	A program during school hours at school.	91 (19.1)

## LIST OF ABBREVIATIONS

WHO: World Health Organization
BMI: Body Mass index
CDC: Centers of Disease Control and Prevention
